# Number 2 Feibi Recipe Inhibits H_2_O_2_-Mediated Oxidative Stress Damage of Alveolar Epithelial Cells by Regulating the Balance of Mitophagy/Apoptosis

**DOI:** 10.3389/fphar.2022.830554

**Published:** 2022-03-17

**Authors:** Xiaofeng Gu, Qi Long, Wan Wei, Jiahuan Tong, Zhipeng Li, Zhengju Zhang, Yang Jiao

**Affiliations:** ^1^ Graduate School, Beijing University of Chinese Medicine, Beijing, China; ^2^ Department of Respiratory and Critical Care Medicine, Chongqing Traditional Chinese Medicine Hospital, Chongqing, China; ^3^ Department of Geriatrics, Dongfang Hospital Affiliated to Beijing University of Chinese Medicine, Beijing, China; ^4^ Department of Respiratory, Zhejiang Provincial Hospital of Chinese Medicine, Hangzhou, China; ^5^ Department of Respiratory, Dongfang Hospital Affiliated to Beijing University of Chinese Medicine, Beijing, China

**Keywords:** number 2 feibi recipe, oxidative stress, alveolar epithelial cells, mitophagy, apoptosis

## Abstract

Reactive oxygen species (ROS)-mediated alveolar epithelial cell (AEC) injury and apoptosis are considered to be the initiating link of idiopathic pulmonary fibrosis (IPF), and protecting AECs can alleviate IPF. This study aimed to explore the protective effect of number 2 Feibi recipe (FBR-2) medicated serum on H_2_O_2_-mediated oxidative stress injury in AECs and further explore its mechanism. We found that FBR-2 can regulate downstream antioxidant enzymes expression by activating nuclear factor erythroid 2-related factor 2 (Nrf2), reducing the level of intracellular ROS, protecting mitochondrial function and improving cell survival. FBR-2 can also activate mitophagy through the PINK1/Parkin pathway. Moreover, FBR-2 can inhibit apoptosis by blocking the mitochondrial apoptosis mechanism. In summary, these data indicate that FBR-2 medicated serum can inhibit H_2_O_2_-mediated oxidative stress damage in AECs by regulating the balance of mitophagy/apoptosis. This study provides new evidence for the antifibrotic effect of FBR-2 and provides new drug candidates for the clinical treatment of IPF.

## Introduction

Idiopathic pulmonary fibrosis (IPF) is a special type of unexplained, chronic, progressive, and fibrotic interstitial pneumonia that occurs in adults and is confined to the lungs (Raghu et al., 2018). The main pathological features of IPF are persistent alveolar epithelial cell (AEC) damage and abnormal proliferation and activation of fibroblasts (Wang and Tang, 2020). The incidence of IPF has risen over time, and in Asia, it is estimated to range between 0.5 and 4.2 cases per 100,000 people per year. The median survival period of IPF is 2–4 years (Lederer and Martinez, 2018), and lung transplantation is the only intervention that has been proven to extend life expectancy (George et al., 2019). The pathogenesis of IPF is not clear, but it is currently believed that reactive oxygen species (ROS)-mediated AEC damage and apoptosis are considered to be the initiating link of IPF. Therefore, drugs and mechanisms used to protect AECs from oxidative stress damage have become the focus of IPF prevention and treatment.

Mitochondria are the main production sites of endogenous ROS and the direct target of ROS oxidative damage (Filomeni et al., 2015). Under normal circumstances, functional mitochondria can regulate the generation and elimination of ROS through detoxification mechanisms. However, when the oxidation/antioxidant balance is broken, excessive ROS can damage mitochondria and promote mitochondrial apoptosis mechanism (Otoupalova et al., 2020). Programmed cell apoptosis can selectively remove aging, damaged or other unwanted cells and maintain a normal cell life cycle. However, the excessive activation of the mitochondrial apoptosis mechanism can cause a large number of ACEs to undergo apoptosis, thereby accelerating the process of IPF (Lee et al., 2018). Mitophagy is a form of selective autophagythat can mark damaged mitochondria for autophagy recognition and degradation (Kubli and Gustafsson, 2012). If mitochondrial autophagy is insufficient or not timely, the damaged mitochondria will produce more pathological ROS, causing a vicious cycle (Ornatowski et al., 2020). Therefore, improving mitochondrial protective autophagy and inhibiting the excessive activation of the mitochondrial apoptosis mechanism may be the key to alleviating the oxidative damage of AECs.

Number 2 Feibi Recipe (FBR-2) is composed of Radix Astragali, Rhodiolae Crenulatae Radix et Rhizoma, Flos Lonicerae Japonicae, Radix Scutellariae, Radix et Rhizoma Salviae Miltiorrhizae, and Radix et Rhizoma Glycyrrhizae at a ratio of 3:3:3:2:2:1. This formula is modified from Feibi Recipe, which is a formula according to Professor Ping’an Zhou’s more than 50 years of clinical experience. Our previous studies have shown that FBR-2 has anti-inflammatory and antioxidant effects (Liu et al., 2018). Moreover, FBR-2 can inhibit pulmonary fibrosis by increasing the expression of antioxidant enzymes and reducing the production of collagen in mouse models (Long et al., 2021). However, the mechanism by which FBR-2 resists oxidative damage is still unclear. In this study, we explored the possible pharmacological effects of FBR-2 by regulating the balance of mitophagy/apoptosis to inhibit H_2_O_2_-mediated oxidative damage, thereby providing a new perspective for the prevention and treatment of IPF.

## Materials and Methods

### Preparation of the FBR-2-Medicated Serum

Forty Sprague**–**Dawley rats were randomly divided into the FBR-2 group (*n* = 15) and the control group (*n* = 25). The rats were housed in a 22 ± 2°C air-conditioned room with a 12 h light-dark cycle and provided with a standard diet with free access to tap water. The rats in the FBR-2 group underwent intragastric administration of FBR-2 (16.3 g/kg) two times a day for 5 days. The rats in the control group received intragastric administration of physiological saline twice a day for 5 days. The volume of intragastric administration of the two groups was consistent. One hour after the last administration, the rats were anesthetized using pentobarbital intraperitoneally. Rat blood samples were collected from the abdominal aorta and centrifuged at 4,000 r/min at 4°C for 15 min. Serum was collected and filtered through a 0.22-μm filter, heat-inactivated at 56°C for 30 min, and stored at −80°C until use.

### Cell Culture and Treatment

Human alveolar epithelial-like cells (A549, Cat No:1101HUM-PUMC000002) were purchased from Cell Resource Center, Institute of Basic Medicine, Chinese Academy of Medical Sciences. The cells were cultured in DMEM/high glucose medium (cat. no. SH30256.01, HyClone, United States) with 10% FBS (cat. no. 12664-025, Gibco, United States), 100 mg/ml streptomycin and 100 U/ml penicillin at 37°C in a humidified 5% CO_2_ incubator. Cells in the exponential growth phase were used in all experiments. A549 cells were treated with 0.6 mM H_2_O_2_ and cocultured with FBR-2-medicated serum (10%) or SB216763(10 μM, MCE, United States) for 24 h. SB216763 is an inhibitor of GSK-3*β*, which can promote autophagy and inhibit apoptosis (Dai et al., 2021). Therefore, SB216763 was selected as the positive control in this study. To ensure uniformity, the total score of serum in all groups was strictly adjusted to 10% with blank serum.

### Cell Proliferation Assay

A549 cells (8 × 10^3^ cells/well) were seeded in 96-well plates. After adherence, the cells were incubated in basal medium (without FBS) with or without H_2_O_2_, FBR-2-medicated serum and SB216763. After 24 h of incubation, 10 μL of CCK-8 (Dojindo, Japan) solution and 90 μL of basal medium were added to each well, and the cells were further incubated in a CO_2_ incubator at 37°C for 1.5 h. The absorbance was measured at 450 nm using an Automatic Microplate Reader (Synergy H1, BioTek, United States).

### Reactive Oxygen Species and Mitochondrial Membrane Potential

The production of ROS was measured by an ROS assay kit (Cat No. S0033S, Beyotime, China). A549 cells were incubated with 10 μM DCFH-DA for 20 min at 37°C in the dark. Then, the cells were gently washed 3 times with warm buffer. The cells were observed under a fluorescence microscope, and the fluorescence intensity was measured at the excitation/emission maxima at 488/525 nm using a fluorescence microplate reader.

MMPs were assessed using the Mitochondrial Membrane Potential Assay Kit with JC-1 (Cat No. C2006, Beyotime, China). After experimentation, the cells were treated with JC-1 staining working solution in a dark environment for 20 min and washed twice with cold staining buffer. A fluorescence microplate reader was used to detect JC-1 monomers at 490/530 nm and JC-1 aggregates at 525/590 nm.

### Determination of MDA, SOD, CAT and GSH-PX

A549 cells (2 × 10^5^ cells/well) were seeded in 6-well plates (2.5 ml/well). After incubation, the cells were collected and the protein concentration was determined by the BCA method. The production of MDA, SOD, CAT and GSH-PX was detected by MDA, SOD, CAT and GSH-PX Assay Kits (Nanjing Jiancheng Bioengineering Institute, China). The operation process was carried out in accordance with the instructions.

### ADP/ATP Ratio

A549 cells (8 × 10^3^ cells/well) were seeded in white opaque microplates. After incubation, the ADP/ATP ratio was detected according to the instructions of the ADP/ATP Ratio Assay kit (Cat No: KA1673, Abnova, United States).

### Fluorescence Colocalization

After 24 h of treatment, Mito-Tracker Red CMXRos (100 nM) working solution was added and incubated at 37°C for 15 min in the dark. Mito-Tracker Red staining working solution was aspirated, Lyso-Tracker Green (75 nM) working solution was added, and the cells were incubated at 37°C for 60 min in the dark. The cells were observed on a fluorescence microscope as soon as possible.

### Immunofluorescence

After 24 h of treatment, Mito-Tracker Red CMXRos (100 nM) working solution was added and incubated at 37°C for 15 min. Then A549 cells were fixed with 4% paraformaldehyde for 20 min, followed by three washes with DPBS. Cells were permeabilized with 0.5% Triton-X 100 for 15 min and blocked with 10% goat serum for 1 h at room temperature. After blocking, the cells were incubated with Cyt C (1: 100, Cat No. ab133504, Abcam, United States) antibody overnight at 4°C. Following incubation with FITC secondary antibody (1:200) for 2 h in the dark, anti-fluorescence quenching mounting solution (including DAPI) was added to the glass bottom dish. Next, the cells were observed under a confocal microscope (FV1000, Olympus, Japan).

### RT–PCR

Total cell RNA was extracted using TRIzol® (Ambion, United States) according to the manufacturer’s instructions. A One Step TB Green PrimescriptTM RT**–**PCR Kit Ⅱ (Cat No. RR086A, TaKaRa, Japan) was used for cDNA synthesis and RT**–**PCR. The reaction conditions were 42°C for 5 min, 95°C for 10 s and then 40 cycles of 95°C for 5 s and 60°C for 31 s. The primers used for this experiment were designed as follows: Nrf2, forward, 5′-TTC​CCG​GTC​ACA​TCG​AGA​G-3′, reverse, 5′-TCC​TGT​TGC​ATA​CCG​TCT​AAA​TC-3′; HO-1, forward, 5′-AAG​ACT​GCG​TTC​CTG​CTC​AAC-3′, reverse, 5′-AAA​GCC​CTA​CAG​CAA​CTG​TCG-3′; PINK1, forward, 5′-GGA​GGA​GTA​TCT​GAT​AGG​GCA​G-3′, reverse, 5′-AAC​CCG​GTG​CTC​TTT​GTC​AC-3′; Parkin, forward, 5′-GTG​TTT​GTC​AGG​TTC​AAC​TCC​A-3′, reverse, 5′-GAA​AAT​CAC​ACG​CAA​CTG​GT C-3′; LC3B, forward, 5′-TTC​AGG​TTC​ACA​AAA​CCC​GC-3′, reverse, 5′- TCT​CAC​ACA​GCC​CGT​TTA​CC-3′; HK-2, forward, 5′-GAG​CCA​CCA​CTC​ACC​CTA​CT-3′, reverse, 5′- CCA​GGC​ATT​CGG​CAA​TGT​G -3′; XIAP, forward, 5′-CCA​GGC​ATT​CGG​CAA​TGT​G-3′, reverse, 5′- TGG​GGT​TAG​GTG​AGC​ATA​GTC -3′; Bcl-2, forward, 5′-CTG​TGG​ATG​ACT​GAG​TAC​CT-3′, reverse, 5′-AGC​CAG​GAG​AAA​TCA​AAC​AGA​G -3′; Bax, forward, 5′-TAC​TTT​GCC​AGC​AAA​CTG​GT-3′, reverse, 5′- TGG​AGA​CAG​GGA​CAT​CAG​TC-3′; ACTB, forward, 5′-TGG​CAC​CCA​GCA​CAA​TGA​A-3′, reverse, 5′-CTA​AGT​CAT​AGT​CCG​CCT​AGA​AGC​A-3′. The primers were synthesized by Shanghai Shenggong Biology Engineering Technology Service, Ltd. All experimental procedures were performed in accordance with the manufacturer’s protocols. The expression of mRNA was analyzed by the 2^−ΔΔCT^ method.

### Western Blot

A549 cells were lysed with RIPA buffer and protease inhibitor. The protein concentrations were measured with the BCA method. Equivalent amounts of total protein were separated by SDS–PAGE and transferred to PVDF membranes. Nonspecific binding was blocked with 5% nonfat milk for 1 h and then incubated overnight at 4°C with antibodies against PINK1 (1: 500, Cat No. 23274-1-AP, Proteintech), Parkin (1: 500, Cat No. 14060-1-AP, Proteintech), LC3B (1: 1,000, Cat No. ab48394, Abcam), HK-2 (1: 2000, Cat No. 22029-1-AP, Proteintech), XIAP (1: 500, Cat No. 10037-1-Ig, Proteintech), Bcl-2 (1: 1,000, Cat No. ab196495, Abcam), Bax (1: 4,000, Cat No. 50599-2-Ig, Proteintech), and *β*-actin (1: 50,000, AC026, Abclonal). All membranes were washed with TBST three times and incubated for 1 h at room temperature with a secondary antibody (1: 10,000, Cat No. AS014, Abclonal). Finally, the membranes were washed three times with TBST. The blots were then developed using an ECL detection kit. The developed blots were subjected to grayscale analysis by ImageJ (NIH, United States) and normalized to an internal control.

### Statistical Analysis

All results were expressed as the means ± standard error of the mean. GraphPad Prism 8.0 software (GraphPad Software, United States) was used for the statistical analyses. One-way analysis of variance (ANOVA) with a post hoc Tukey multiple comparison test was used to analyze the data between different groups. *p* < 0.05 was considered statistically significant.

## Results

### FBR-2-Medicated Serum Inhibit H_2_O_2_-Induced Injury in A549 Cells

**FIGURE 1:**
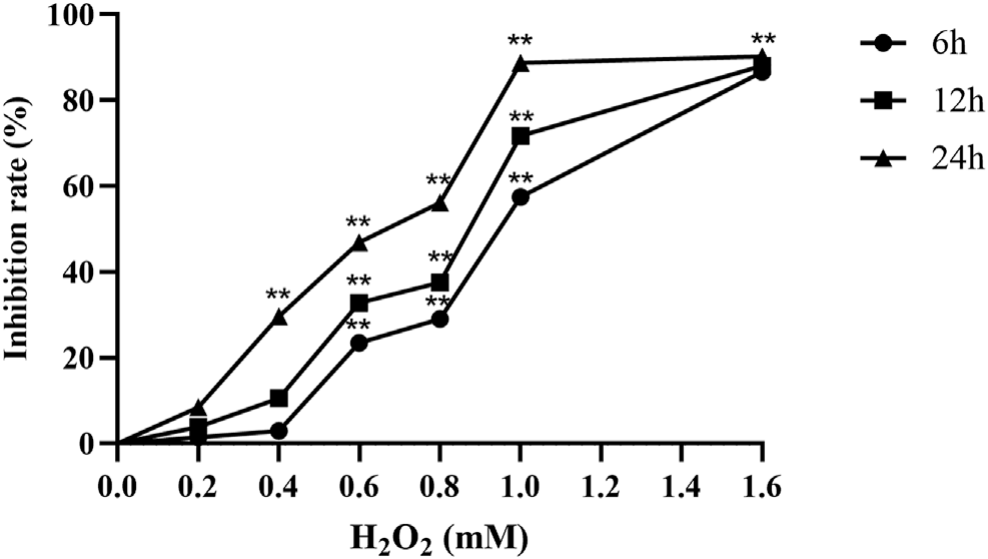
H_2_O_2_ inhibits the proliferation of A549 cells (*n* = 6). **p < 0.01, compared with the control group.

A549 cells were treated with 0–1.6 mM H_2_O_2_ (6, 12 and 24 h). The results showed that the inhibitory effect of H_2_O_2_ on the proliferation of A549 cells was positively correlated with the time-dose. When 0.6 mM H_2_O_2_ was added to the cells for 24 h, the proliferation inhibition rate of A549 cells was 46.84% (*p* < 0.01). Therefore, we chose 0.6 mM H_2_O_2_ to intervene for 24 h as the concentration and time of the H_2_O_2_-mediated A549 cell oxidative damage model (Figure 1).

**FIGURE 2:**
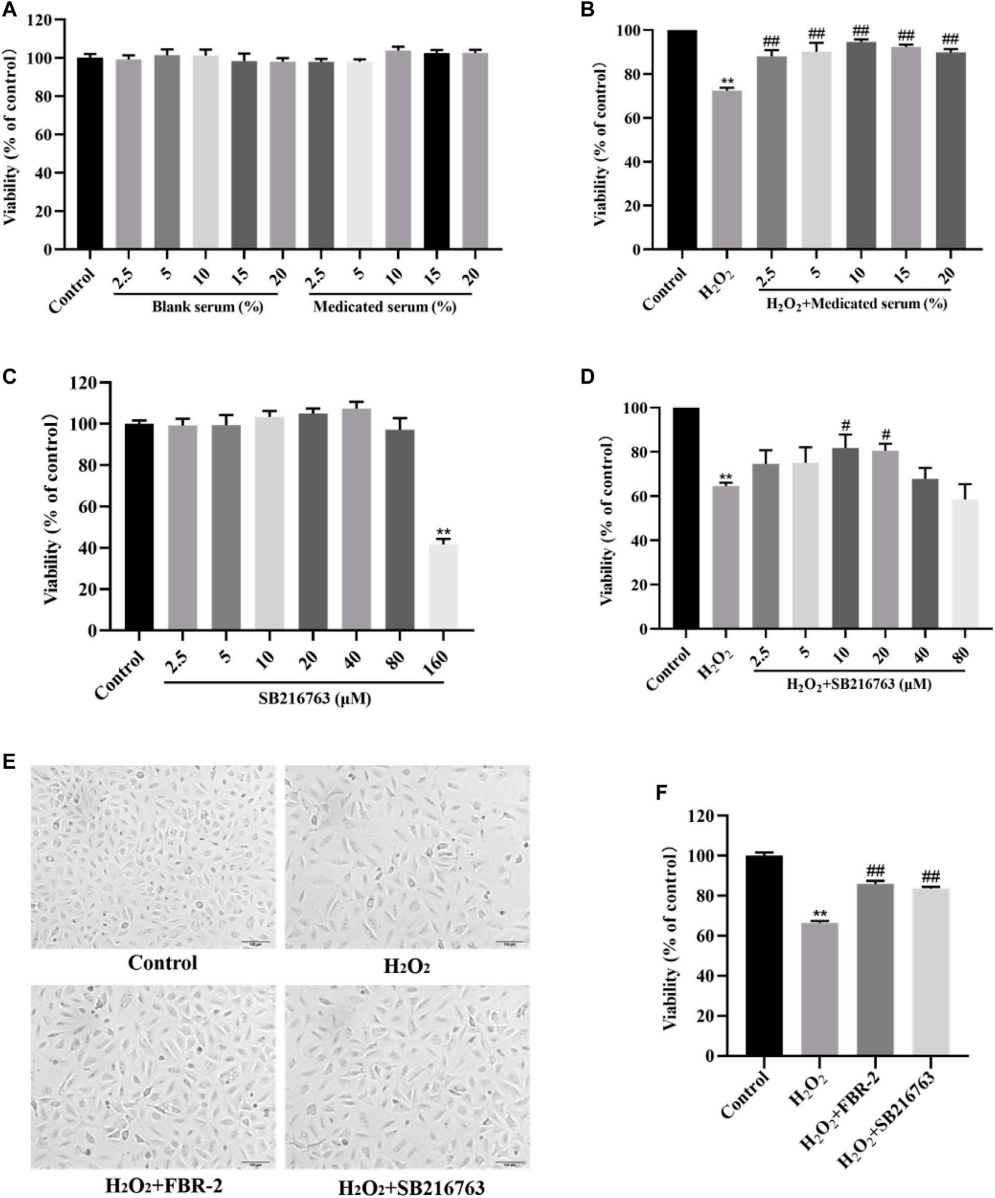
FBR-2 protects A549 cells from oxidative stress damage. **(A)** The effects of blank serum and FBR-2-medicated serum on the viability of A549 cells (*n* = 6). **(B)** The effect of FBR-2-medicated serum on the viability of A549 cells induced by H_2_O_2_ (*n* = 6). **(C)** The effect of SB216763 on the viability of A549 cells (*n* = 6). **(D)** The effect of SB216763 on the viability of A549 cells induced by H_2_O_2_ (*n* = 6). **(E)** The effect of FBR-2-medicated serum on the morphology of A549 cells induced by H_2_O_2_. **(F)** The protective effect of FBR-2 on A549 cells induced by H_2_O_2_ (*n* = 6). **p < 0.01, compared with the control group; ^*#*^ *p* < 0.05 and ^#*#*^ *p* < 0.01, compared with the H_2_O_2_ group.

To evaluate the effect of FBR-2-medicated serum and blank serum on A549 cells, we treated A549 cells with a volume fraction of 0–20% medicated serum and blank serum for 24 h. The results showed that compared with the blank control group, there was no significant difference in the survival rate of the cells in each group (*p* > 0.05), which showed that when the volume fraction of the medicated serum and the blank serum were in the range of 2.5–20%, there was no toxic effect on A549 cells (Figure 2A). Therefore, we selected different volume fractions of medicated serum and cocultured them with 0.6 mM H_2_O_2_ for 24 h. The results showed that compared with the H_2_O_2_ group, the medicated serum in each experimental group could significantly increase the cell viability level (*p* < 0.01), and when the volume fraction was 10%, the medicated serum had the greatest promotion effect (Figure 2B). Therefore, 10% volume fraction was selected as the optimal intervention concentration of FBR-2 medicated serum.

Similarly, we treated A549 cells with SB216763 at a concentration of 0–160 μM for 24 h. The results showed that the concentration of SB216763 had no significant toxic effect on A549 cells in the range of 2.5–80 μM (*p >* 0.05), while 160 μM reduced cell viability (*p* < 0.01) (Figure 2C). Therefore, we selected SB216763 in the concentration range of 2.5–80 μM and cocultured it with 0.6 mM H_2_O_2_ for 24 h. The results showed that compared with the H_2_O_2_ group, when the concentration was 10 or 20 μM, SB216763 increased cell viability (*p* < 0.01), and the survival rate was highest at 10 μM (Figure 2D). Excluding the possible damage caused by high concentrations, 10 μM was selected as the best intervention concentration of SB216763.

The cells of the control group were spindle-shaped and closely arranged, and the cells grew in a good state. The cells in the H_2_O_2_ group were sparsely arranged, and some of the cells had incomplete structures. Suspended cells and cell debris were observed, and the survival rate was reduced (*p* < 0.01). Compared with the H_2_O_2_ group, the suspended cells and cell debris in the FBR-2 group and the SB216763 group were reduced, and the survival rate was significantly improved (*p* < 0.01) (Figures 2E,F).

### FBR-2 Inhibits the Production of Reactive Oxygen Species and MDA in Cells Induced by H_2_O_2_

**FIGURE 3:**
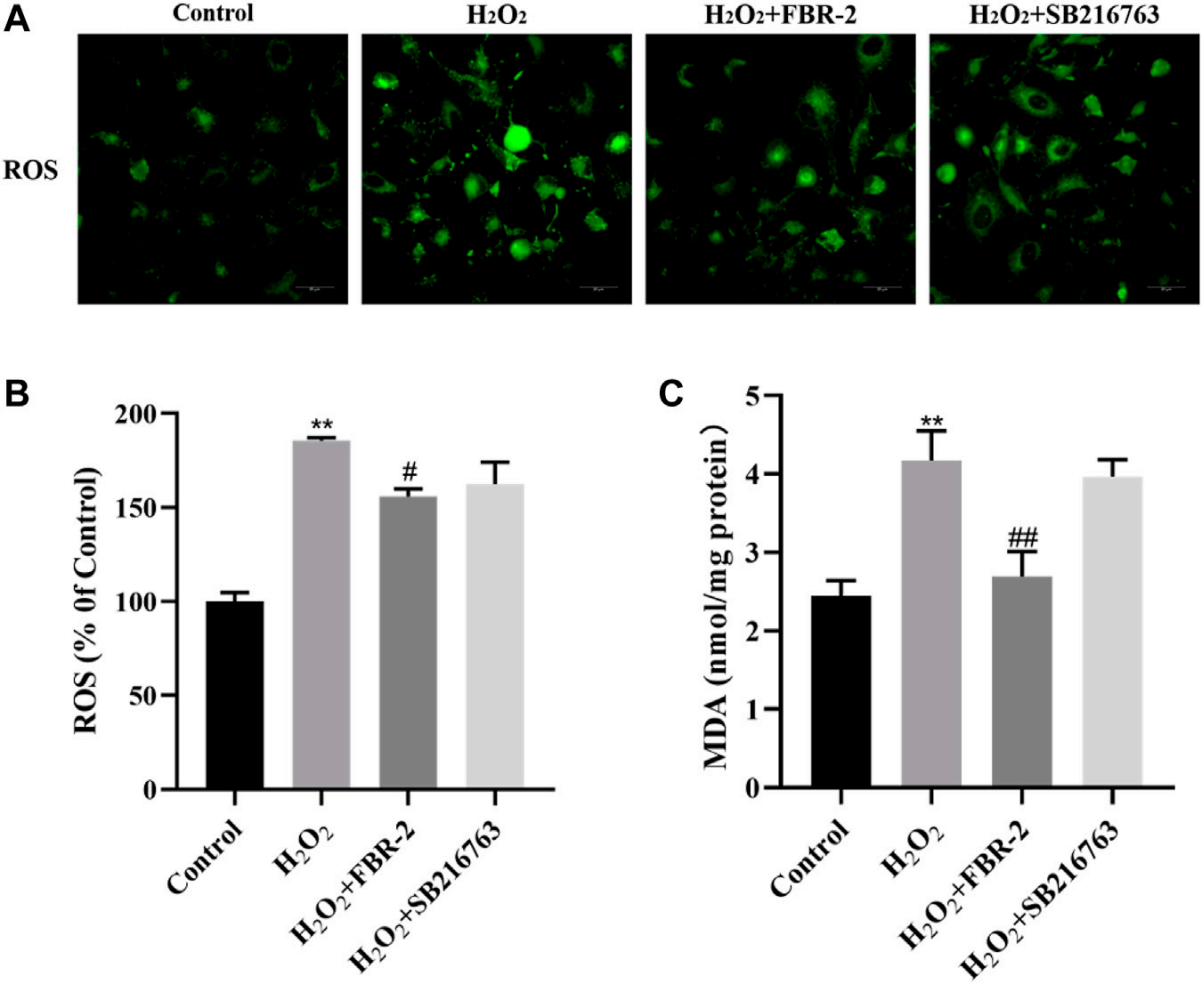
FBR-2 inhibits the production of ROS and MDA in A549 cells induced by H_2_O_2_. **(A)** Fluorescence microscopy observation of ROS production in cells. **(B)** The level of ROS (*n* = 5). **(C)** The level of MDA (*n* = 6). **p < 0.01, compared with the control group; ^#^ *p* < 0.05 and ^##^ *p* < 0.01, compared with the H_2_O_2_ group.

Intracellular ROS play an important role in oxidative stress-mediated cell damage, and MDA is the end product of lipid peroxidation, which can indirectly reflect the degree of oxidative damage. The results showed that H_2_O_2_ increased the levels of ROS and MDA in A549 cells (*p* < 0.01), while FBR-2 medicated serum significantly reduced the production of ROS and MDA (*p* < 0.01 or *p* < 0.05) (Figure 3).

### FBR-2 Protects Cells by Regulating Enzyme Activity

**FIGURE 4:**
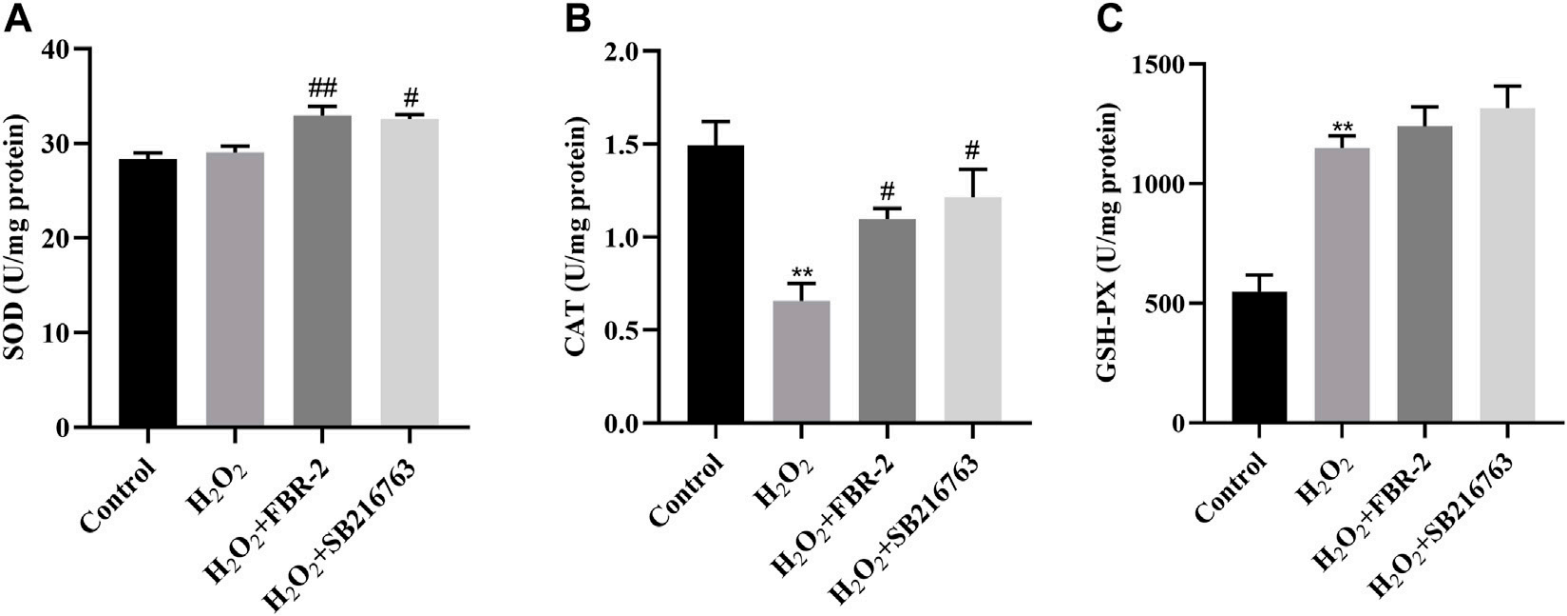
FBR-2 protects cells by regulating enzyme activity. **(A)** Activity of SOD (*n* = 6). **(B)** Activity of CAT (*n* = 6). **(C)** Activity of GSH-PX (*n* = 6). **p < 0.01, compared with the control group; ^#^ *p* < 0.05 and ^##^ *p* < 0.01, compared with the H_2_O_2_ group.

SOD, CAT and GSH-PX are important components of the enzyme defense system. To determine the effect of FBR-2 medicated serum on H_2_O_2_-treated A549 cells, we tested the levels of SOD, CAT and GSH-PX. The results showed that compared with the control group, the SOD activity of the H_2_O_2_ group was not different (*p* > 0.05), the activity of CAT was decreased (*p* < 0.01), and the activity of GSH-PX was increased (*p* < 0.01). Compared with the H_2_O_2_ group, FBR-2 and SB216763 significantly improved SOD and CAT activity. In terms of GSH-PX activity level, the two groups tended to improve, but the difference was not statistically significant(*p* > 0.05) (Figure 4).

### FBR-2 Increased the Expression of Nrf2 and HO-1 mRNA in A549 Cells

**FIGURE 5:**
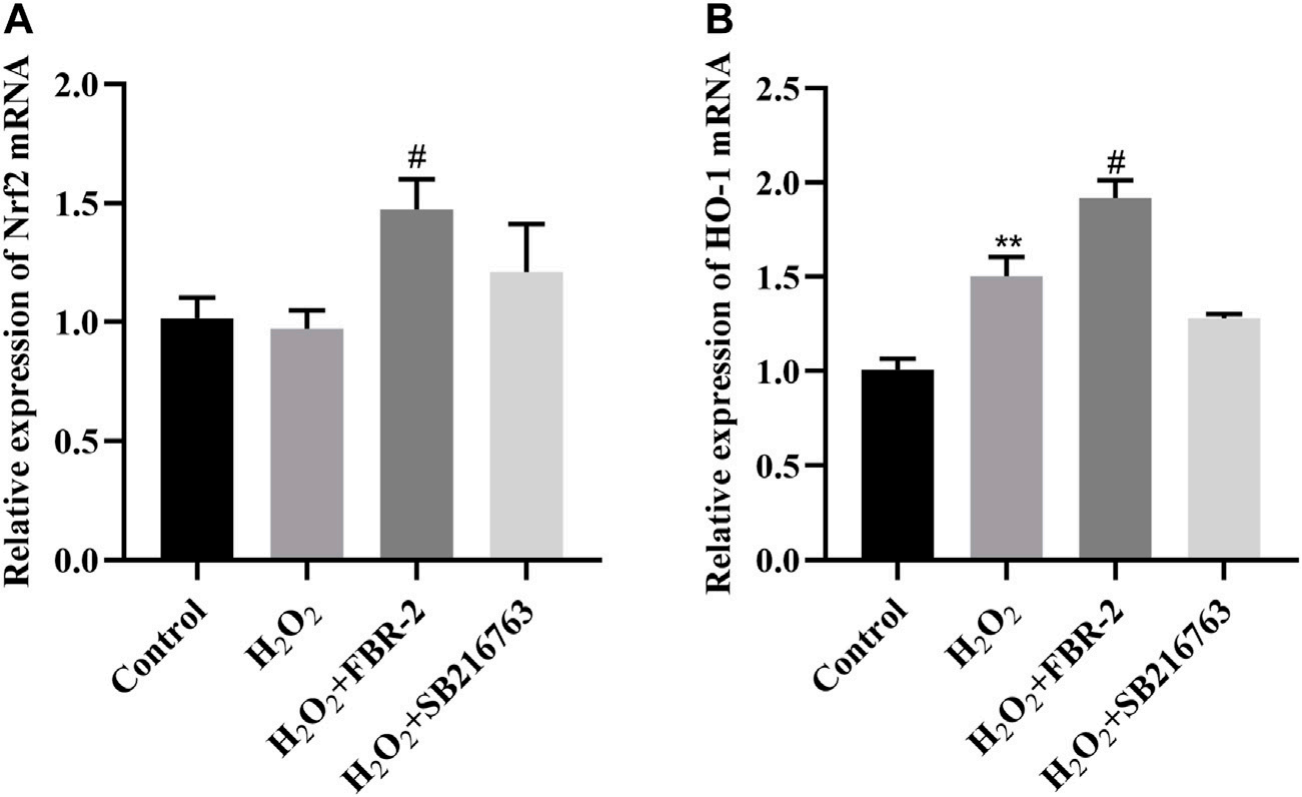
FBR-2 increased the expression of Nrf2 and HO-1 mRNA in A549 cells. **(A)** Nrf2 mRNA levels (*n* = 6). **(B)** HO-1 mRNA levels (*n* = 6). **p < 0.01, compared with the control group; ^#^ *p* < 0.05, compared with the H_2_O_2_ group.

Nuclear factor erythroid 2-related factor 2 (Nrf2) is an important transcription factor for the antioxidative stress response and drug detoxification. It can regulate the transcription of a variety of detoxification enzymes including HO-1 to maintain the redox balance in the cell (He et al., 2020). Compared with the control group, the expression of Nrf2 mRNA in the H_2_O_2_ group was slightly lower (*p* > 0.05), and the expression of HO-1 mRNA was increased (*p* < 0.01). Compared with the H_2_O_2_ group, the expression of Nrf2 and HO-1 mRNA in the FBR-2 group was significantly increased (*p* < 0.05). The expression of Nrf2 mRNA in the SB216763 group increased, and the expression of HO-1 mRNA decreased, but the difference was not statistically significant (*p* > 0.05) (Figure 5).

### FBR-2 Reduced the Effect of H_2_O_2_ on the Mitochondrial Function of A549 Cells

**FIGURE 6:**
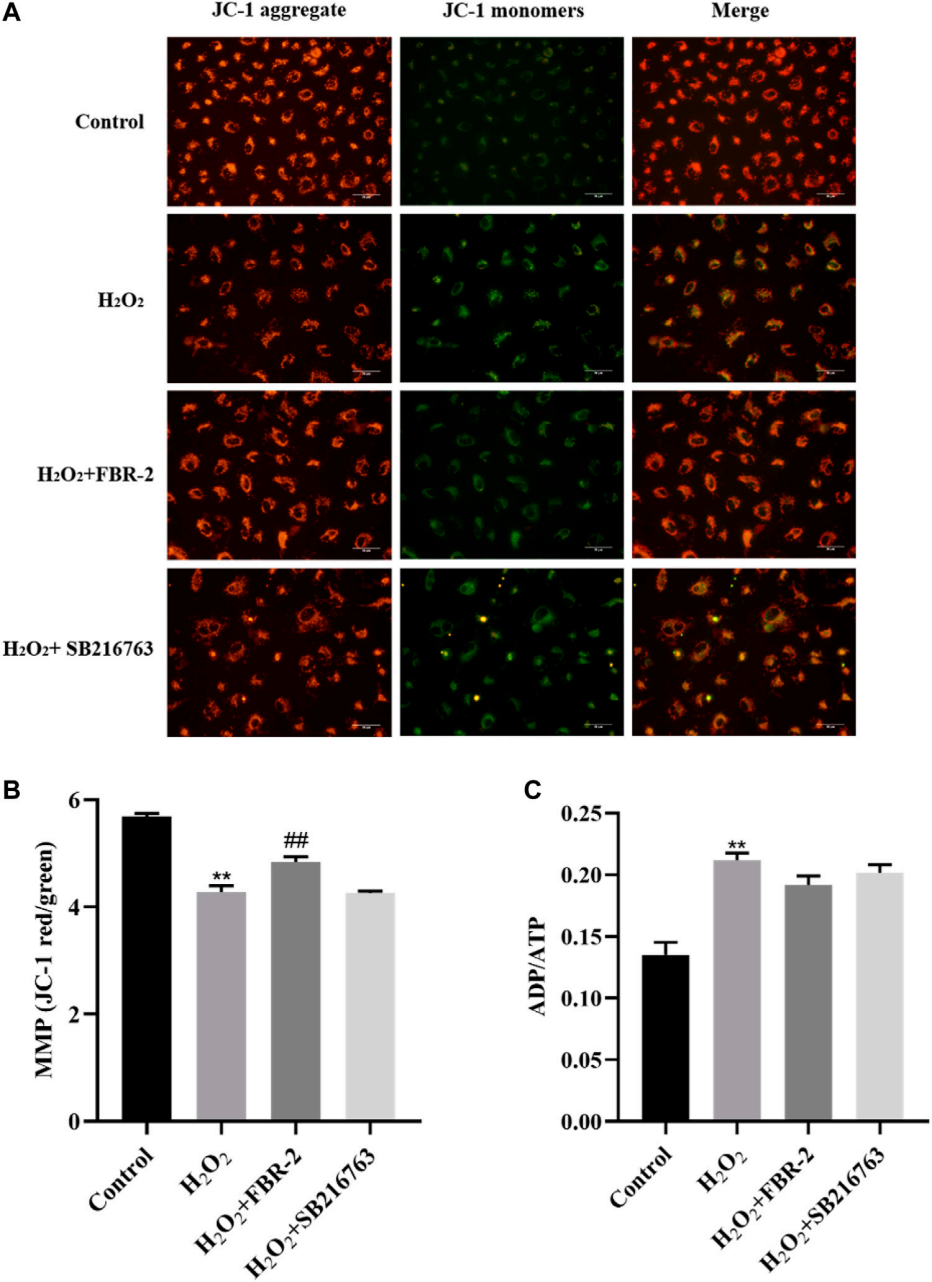
Effect of FBR-2 on MMP and ADP/ATP ratio of A549 cells induced by H_2_O_2_. **(A)** Fluorescence microscopy observation of MMP. **(B)** The level of MMP (*n* = 5). **(C)** ADP/ATP ratio (*n* = 6). **p < 0.01, compared with the control group; ^##^ *p* < 0.01, compared with the H_2_O_2_ group.

In order to clarify the effect of FBR-2 on mitochondrial function, we further tested the level of MMP and the ratio of ADP/ATP. The results showed that H_2_O_2_ could downregulate the level of MMP (*p* < 0.01), while FBR-2 could attenuate this effect (*p* < 0.01). Compared with the control group, the ADP/ATP ratio of the H_2_O_2_ group was significantly higher (*p* < 0.01). Compared with the H_2_O_2_ group, the FBR-2 group and the SB216763 group decreased, but the difference was not statistically significant (*p* > 0.05) (Figure 6).

### FBR-2 Promotes Mitophagy

**FIGURE 7:**
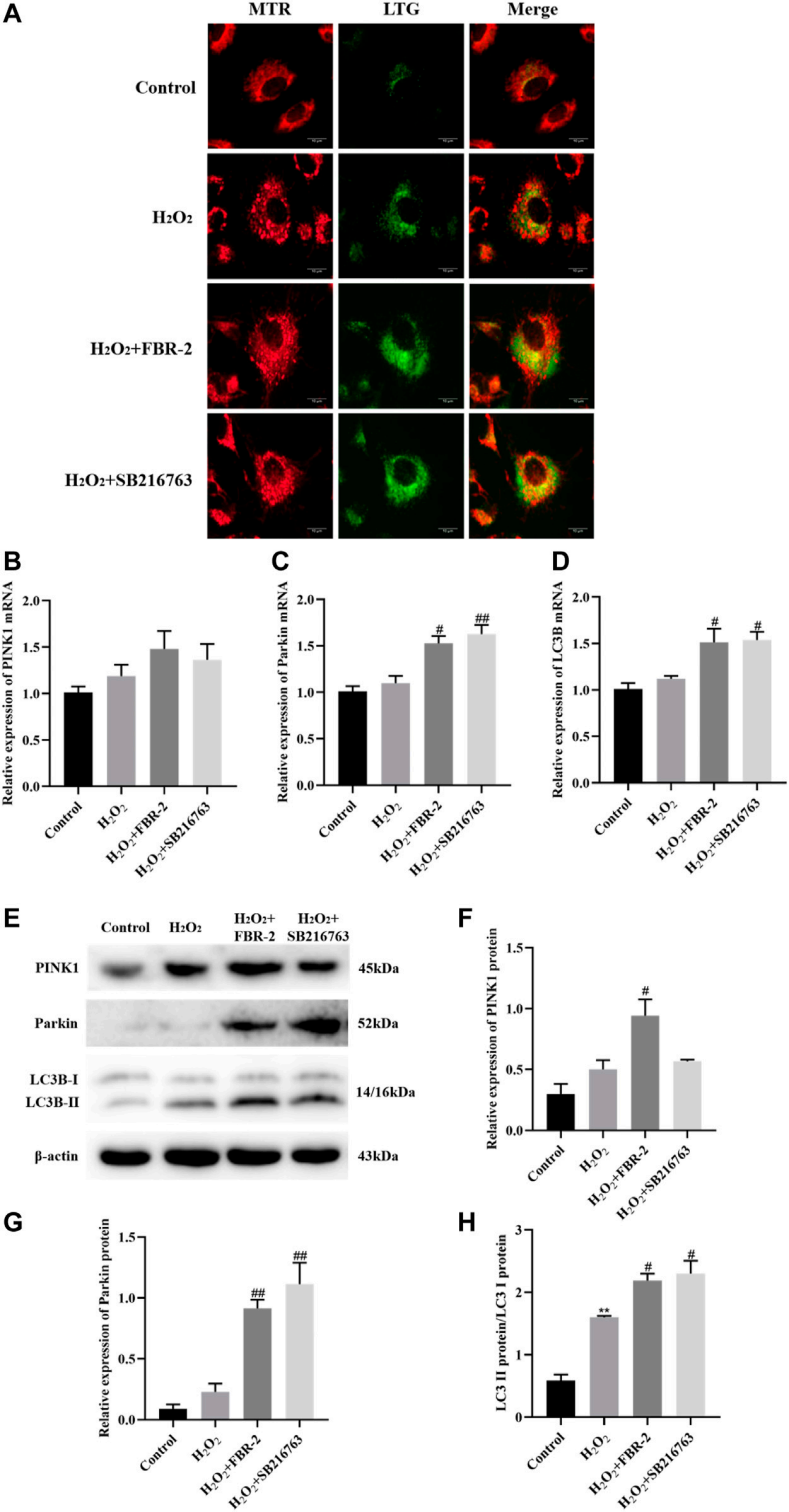
FBR-2 promotes mitophagy. **(A)** Fluorescence colocalization between mitochondria (MTR) and lysosomes (LTG). **(B)** PINK1 mRNA levels (*n* = 6). **(C)** Parkin mRNA levels (*n* = 6). **(D)** LC3B mRNA levels (*n* = 6). **(E)** The blots of PINK1, Parkin, LC3B and *β*-actin. **(F)** PINK1 protein levels (*n* = 3). **(G)** Parkin protein levels (*n* = 3). **(H)** The ratio of LC3B II/I (*n* = 3). **p < 0.01, compared with the control group; ^#^ *p* < 0.05 and ^##^ *p* < 0.01, compared with the H_2_O_2_ group.

To determine whether mitophagy is involved in the protective effect of FBR-2 on H_2_O_2_-treated A549 cells, we further tested mitophagy. In this study, we used fluorescent probes to colocalize mitochondria (red) and lysosomes (green) to observe the occurrence of mitophagy. The results showed that the control group had almost no colocalization (yellow dots). Mitochondrial fluorescence in the H_2_O_2_ group was scattered, lysosomal fluorescence was enhanced, and scattered colocalization points were observed. Compared with the H_2_O_2_ group, the level of colocalization in the FBR-2 group and the SB216763 group was significantly enhanced (Figure 7A).

The PINK1/Parkin pathway is the main mechanism of mitophagy, which can recruit autophagosomes to damaged mitochondria. When autophagosomes are formed, the cytoplasmic protein LC3B-I is converted to LC3B-II by enzymatic hydrolysis. Therefore, the increase in the LC3B-II/I ratio is a sign of the beginning of autophagy. The results showed that compared with the control group, the expression of PINK1, Parkin and LC3B mRNA in the H_2_O_2_ group increased slightly, but the difference was not statistically significant (*p* > 0.05). Compared with the H_2_O_2_ group, the expression of Parkin and LC3B mRNA was significantly higher in the FBR-2 group and the SB216763 group (*p* < 0.05 or *p* < 0.01) (Figures 7B–D).

The protein expression was close to that of mRNA. Compared with that in the control group, the LC3B-II/I ratio in the H_2_O_2_ group was significantly higher (*p* < 0.01). Compared with the H_2_O_2_ group, the expression of PINK1and Parkin and the ratio of LC3B-II/I in the FBR-2 group were significantly increased (*p* < 0.01), and the expression of Parkin and the ratio of LC3B-II/I in the SB216763 group were also significantly increased. (*p* < 0.01) (Figures 7E–H).

### FBR-2 Inhibits Cell Apoptosis

**FIGURE 8:**
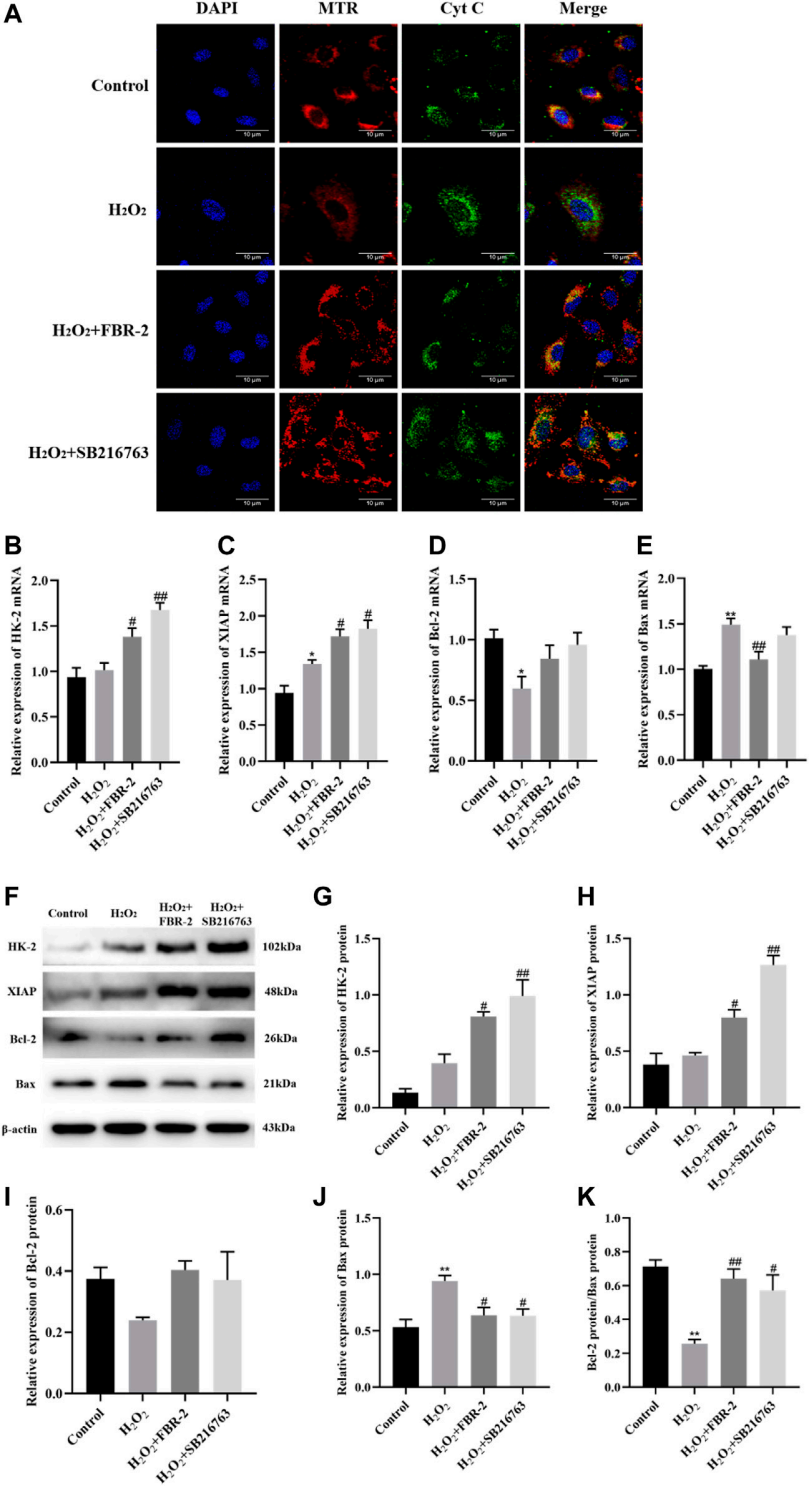
FBR-2 inhibits cell apoptosis. **(A)** Immunofluorescence colocalization between mitochondria (MTR) and Cyt C. **(B)** HK-2 mRNA levels (*n* = 6). **(C)** XIAP mRNA levels (*n* = 6). **(D)** Bcl-2 mRNA levels (*n* = 6). **(E)** Bax mRNA levels (*n* = 6). **(F)** The blots of HK-2, XIAP, Bcl-2, Bax and *β*-actin. **(G)** HK-2 protein levels (*n* = 3). **(H)** XIAP protein levels (n = 3). **(I)** Bcl-2 protein levels (*n* = 3). **(J)** Bax protein levels (*n* = 3). **(K)** The ratio of Bcl-2/Bax. *p < 0.05 and **p < 0.01, compared with the control group; ^#^ *p* < 0.05 and ^##^ *p* < 0.01, compared with the H_2_O_2_ group.

Subsequently, we explored the effect of FBR-2 on the mechanism of mitochondrial apoptosis. The release of cytochrome C (Cyt C) is the main step of the endogenous apoptosis mechanism. Under physiological conditions, Cyt C is located in the protein and lipid complex of the inner mitochondrial membrane. During oxidative stress, Cyt C can sense the decrease in MMP, release from mitochondria to the cytoplasm, and mediate cell apoptosis. Studies have shown that in the control group, mitochondria (red) and Cyt C (green) have obvious colocalization (yellow dots) and surround the nucleus. There was almost no colocalization in the H_2_O_2_ group, and the green fluorescence increased and was distributed loosely, indicating that Cyt C had been released from the mitochondria into the cytoplasm. Mitochondria and Cyt C were loosely distributed in the FBR-2 and SB216763 groups, but there was significant colocalization (Figure 8A).

HK-2, XIAP and Bcl-2 are apoptosis-inhibiting proteins, and Bax is one of the main pro-apoptotic proteins. The ratio of Bcl-2/Bax is usually used to measure whether a cell is undergoing apoptosis. The results showed that in terms of mRNA expression, compared with the control group, the expression of XIAP in the H_2_O_2_ group was irritably increased (*p* < 0.01), the expression of Bcl-2 was significantly reduced (*p* < 0.05), and the expression of Bax was significantly increased (*p* < 0.01). Compared with the H_2_O_2_ group, the FBR-2 group and the SB216763 group had a significant increase in the expression of HK-2 and XIAP (*p* < 0.05 or *p* < 0.01), but there was no statisticant difference in the expression of Bcl-2. In addition, the FBR-2 group significantly reduced the expression of Bax (*p* < 0.01) (Figures 8B–E).

In terms of protein expression, treatment with H_2_O_2_ significantly upregulated the expression of Bax in A549 cells and significantly downregulated the ratio of Bcl-2/Bax. Compared with the H_2_O_2_ group, FBR-2 and SB216763 promoted the expression of HK-2 and XIAP (*p* < 0.05 or *p* < 0.01), and downregulated the expression of Bax (*p* < 0.05). In addition, the ratio of Bcl-2/Bax also increased significantly (*p* < 0.05 or *p* < 0.01) (Figures 8F–K).

## Discussion

The pathogenesis of IPF is very complex, but it is generally believed that the injury and dysfunction of AECs are the initiating link of IPF. Many studies have confirmed that the apoptosis level of pulmonary AECs in patients with IPF is increased, and this phenomenon is not seen in the nonfibrotic lung (Uhal et al., 1998; Barbas-Filho et al., 2001). ROS-mediated oxidative stress is the main cause of AEC injury and apoptosis. Exogenous ROS mainly come from environmental toxins, including cigarette smoke, asbestos, zinc oxide and other substances. They can break through the defense of the upper respiratory tract and directly contact the alveolar epithelium, resulting in excessive production of ROS in AECs (Cheresh et al., 2013). Endogenous ROS are mainly byproducts of the mitochondrial electron transfer chain (ETC) during oxidative phosphorylation and ATP production (Otoupalova et al., 2020). The excessive production of ROS and/or the reduction of local antioxidant defense cause the imbalance of ROS, which mediates the oxidative stress damage of the body, thus damaging protein, lipid and mitochondrial DNA (Dunn et al., 2015), and finally promote the mechanism of mitochondrial apoptosis and cell apoptosis. While AECs undergo apoptosis, immune cells such as alveolar macrophages and lymphocytes are activated and produce a variety of cytokines, including TGF-*β*1, which drives the development of IPF and promotes further apoptosis of AECs (Rangarajan et al., 2017; Liu et al., 2019). Our study shows that FBR-2 can reduce the level of ROS in A549 cells mediated by H_2_O_2_, reduce oxidative stress injury and improve the cell survival rate.

To inhibit oxidative stress, there are a series of detoxification mechanisms, including nonenzymatic (nonspecific) and enzymatic (specific) mechanisms. The enzyme defense mechanism plays an important role in the process of antioxidation. Superoxide dismutase (SOD), catalase (CAT), glutathione peroxidase (GSH-PX), heme oxygenase (HO) and other antioxidant enzymes can antagonize ROS and maintain cell redox homeostasis (Netto and Antunes, 2016). As an important antioxidant transcription factor, Nrf2 can induce and regulate the expression of direct antioxidant enzymes and phase II detoxification enzymes in cells (Jiang et al., 2021). Nrf2 silencing can reduce the barrier function of alveolar epithelium, and Nrf2 activation can reverse this effect (Kukoyi et al., 2019). Our study shows that FBR-2 can regulate the expression of downstream antioxidant enzymes such as HO-1, SOD and CAT by activating Nrf2, to antagonize ROS and protect mitochondrial function.

Mitophagy is a process of selective degradation of mitochondriathat can remove damaged mitochondria and prevent the excessive production of mtROS. There is evidence that autophagy is insufficient in IPF, and preventing autophagy can lead to epithelial cell aging and increased myofibroblast differentiation (Araya et al., 2013). The PINK1/Parkin-dependent mechanism is currently recognized as a mechanism of mitophagy. PINK1 can quickly sense mitochondrial damage and aggregate and activate on the mitochondrial outer membrane. Activated PINK1 can phosphorylate its key substrates ubiquitin (Ub) and Parkin, leading to the translocation and activation of Parkin (Harper et al., 2018). Then, a feedforward mechanism is formed to induce powerful mitochondrial autophagy and quickly remove damaged mitochondria (Wang et al., 2020). Our research shows that FBR-2 can activate mitochondrial autophagy by promoting the expression of PINK1, Parkin and LC3B-II, and activate the secondary defense of the antioxidant system to protect cells.

Mitochondrial-mediated endogenous apoptosis is the main form of AEC apoptosis. Excessive ROS can open the mitochondrial permeability transition pore (mPTP) and cause the loss of MMP. Subsequently, mitochondria release Cyt C and other proapoptotic proteins, which further activate the downstream caspase cascade, and finally initiate apoptosis (Li et al., 2021). Bcl-2 family proteins are the main regulators of endogenous apoptosis. Breaking the balance between Bcl-2 and Bax will induce the release of Cyt C (Veerasamy et al., 2021). HK2 is a structural protein of mPTP, which is essential for maintaining the integrity of mitochondrial membrane structure and MMP (Duan et al., 2021). As the main negative regulator in the process of mitochondrial apoptosis, XIAP can inhibit apoptosis by blocking the activation of Caspase-3/7/9 (Tu and Costa, 2020). Our research shows that FBR-2 can block the mitochondrial apoptotic pathway and inhibit cell apoptosis by promoting the expression of HK-2 and XIAP and increasing the ratio of Bcl-2/Bax.

Previous studies have suggested that excessive mitophagy can trigger endogenous apoptosis, while inhibition of mitophagy can reduce apoptosis (Gao et al., 2017; Prakash et al., 2021), which seems to contradict our research results. However, more evidence shows that AEC apoptosis caused by mitochondrial dysfunction and mitophagy defects plays a key role in the pathogenesis of pulmonary fibrosis (Bueno et al., 2015; Malsin and Kamp, 2018). Recent studies have further confirmed that the lack of the mitochondrial autophagy related protein PINK1 enhances oxidant induced mtDNA damage and aggravates AEC apoptosis (Kim et al., 2020). Therefore, combined with our research results, we believe that the mechanism of oxidant induced AEC apoptosis may be that insufficient autophagy leads to the failure of timely and efficient clearance of damaged mitochondria, leading to the further accumulation of mtROS and finally triggering the mechanism of mitochondrial apoptosis. Thus, it is very important for the prevention and treatment of IPF to protect against mitophagy and inhibit the mechanism of mitochondrial apoptosis to maintain the balance between mitophagy and apoptosis in ACEs.

## Conclusion

In conclusion, our study confirmed that FBR-2 can inhibit H_2_O_2_-mediated oxidative stress damage inA549 cells by regulating the balance of mitophagy/apoptosis. However, due to the complexity of its components, traditional Chinese medicine compounds have multitarget and multipathway pharmacological characteristics. Therefore, other antifibrosis mechanisms of FBR-2 need to be studied further.

## Data Availability

The raw data supporting the conclusion of this article will be made available by the authors, without undue reservation.
