# Supplementary figures and images for: Number 2 Feibi Recipe Inhibits H2O2-Mediated Oxidative Stress Damage of Alveolar Epithelial Cells by Regulating the Balance of Mitophagy/Apoptosis
Source: Front Pharmacol. 2022 Mar 17;13:830554. doi: 10.3389/fphar.2022.830554 (PMC8968876; doi:10.3389/fphar.2022.830554)

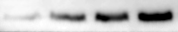

Supplement: Supplementary file 1 [file DataSheet1.ZIP › 4.western blot/All the blots/1.HK-2/HK2-1.tif]

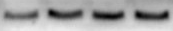

Supplement: Supplementary file 1 [file DataSheet1.ZIP › 4.western blot/All the blots/1.HK-2/HK2-2.tif]

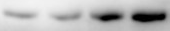

Supplement: Supplementary file 1 [file DataSheet1.ZIP › 4.western blot/All the blots/1.HK-2/HK2-3.tif]

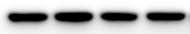

Supplement: Supplementary file 1 [file DataSheet1.ZIP › 4.western blot/All the blots/1.HK-2/β-actin-1.tif]

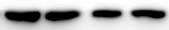

Supplement: Supplementary file 1 [file DataSheet1.ZIP › 4.western blot/All the blots/1.HK-2/β-actin-2.tif]

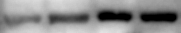

Supplement: Supplementary file 1 [file DataSheet1.ZIP › 4.western blot/All the blots/2.XIAP/XIAP-1.tif]

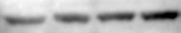

Supplement: Supplementary file 1 [file DataSheet1.ZIP › 4.western blot/All the blots/2.XIAP/XIAP-2.tif]

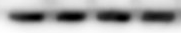

Supplement: Supplementary file 1 [file DataSheet1.ZIP › 4.western blot/All the blots/2.XIAP/XIAP-3.tif]

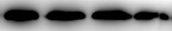

Supplement: Supplementary file 1 [file DataSheet1.ZIP › 4.western blot/All the blots/2.XIAP/β-actin-3.tif]

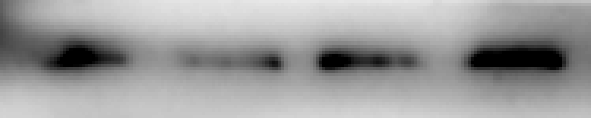

Supplement: Supplementary file 1 [file DataSheet1.ZIP › 4.western blot/All the blots/3.Bcl-2/bcl2-2.tif]

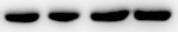

Supplement: Supplementary file 1 [file DataSheet1.ZIP › 4.western blot/All the blots/3.Bcl-2/β-actin-2.tif]

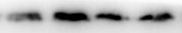

Supplement: Supplementary file 1 [file DataSheet1.ZIP › 4.western blot/All the blots/4.BAX/bax-2.tif]

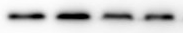

Supplement: Supplementary file 1 [file DataSheet1.ZIP › 4.western blot/All the blots/4.BAX/bax-3.tif]

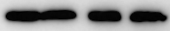

Supplement: Supplementary file 1 [file DataSheet1.ZIP › 4.western blot/All the blots/4.BAX/β-actin-1.tif]

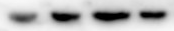

Supplement: Supplementary file 1 [file DataSheet1.ZIP › 4.western blot/All the blots/5.PINK1/PINK1-1.tif]

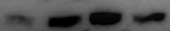

Supplement: Supplementary file 1 [file DataSheet1.ZIP › 4.western blot/All the blots/5.PINK1/PINK1-3.tif]

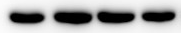

Supplement: Supplementary file 1 [file DataSheet1.ZIP › 4.western blot/All the blots/5.PINK1/β-actin-1.tif]

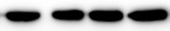

Supplement: Supplementary file 1 [file DataSheet1.ZIP › 4.western blot/All the blots/5.PINK1/β-actin-3.tif]

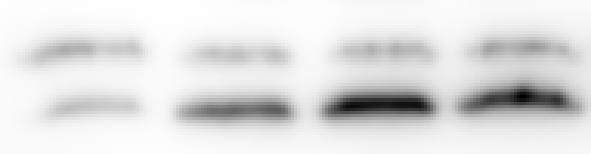

Supplement: Supplementary file 1 [file DataSheet1.ZIP › 4.western blot/All the blots/7.LC-3/LC3-7.gif]

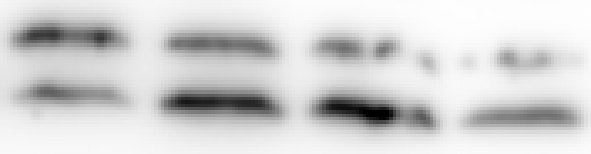

Supplement: Supplementary file 1 [file DataSheet1.ZIP › 4.western blot/All the blots/7.LC-3/LC3-8.gif]
